# A theory of cerebellar learning as spike-based reinforcement learning in continuous time and space

**DOI:** 10.1093/pnasnexus/pgaf302

**Published:** 2025-09-18

**Authors:** Rin Kuriyama, Hideyuki Yoshimura, Tadashi Yamazaki

**Affiliations:** Graduate School of Informatics and Engineering, The University of Electro-Communications, Tokyo 182-8585, Japan; Neural Computation Unit, Okinawa Institute of Science and Technology Graduate University, Okinawa 904-0495, Japan; Graduate School of Informatics and Engineering, The University of Electro-Communications, Tokyo 182-8585, Japan

**Keywords:** cerebellum, reinforcement learning, spiking neural network, computational model, machine learning

## Abstract

The cerebellum has been considered to perform error-based supervised learning via long-term depression (LTD) at synapses between parallel fibers and Purkinje cells (PCs). Since the discovery of multiple synaptic plasticity other than LTD, recent studies have suggested that synergistic plasticity mechanisms could enhance the learning capability of the cerebellum. Indeed, we have proposed a concept of cerebellar learning as a reinforcement learning (RL) machine. However, there is still a gap between the conceptual algorithm and its detailed implementation. To close this gap, in this research, we implemented a cerebellar spiking network as an RL model in continuous time and space, based on known anatomical properties of the cerebellum. We confirmed that our model successfully learned a state value and solved the mountain car task, a simple RL benchmark. Furthermore, our model demonstrated the ability to solve the delay eyeblink conditioning task using biologically plausible internal dynamics. Our research provides a solid foundation for cerebellar RL theory that challenges the classical view of the cerebellum as primarily a supervised learning machine.

Significance Statement The cerebellum has traditionally been understood as a supervised learning (SL) machine, yet its potential for reinforcement learning (RL) remains unexplored. This study presents the first cerebellar spiking network model capable of implementing RL through an actor-critic framework, grounded in known anatomical and functional properties. In this model, Purkinje cells and molecular layer interneurons act as actor and critic, respectively, without explicit temporal difference error computation. The model successfully performs both standard RL tasks, such as the mountain car task, and a cerebellum-dependent motor learning task known as the delay eyeblink conditioning. These results suggest that the cerebellum may integrate both SL and RL mechanisms, which may supersede the classical SL-centric view known as the Marr–Albus–Ito model.

## Introduction

Theories of learning in the cerebellum have historically attracted considerable interest. One of the most widely known theories, known as the Marr–Albus–Ito model (1–3), proposes that the cerebellum acts as an error-based supervised learning (SL) machine (4) to modify output and reduce the discrepancy between actual and desired outputs. In this theory, climbing fibers (CFs) deliver error or teacher signals that can trigger long-term depression (LTD) at parallel fiber (PF) and Purkinje cell (PC) synapses (5). Since the discovery of LTD in the early 1980s (6), biological research has found many other forms of synaptic plasticity in the cerebellum (7). These findings have been extending the Marr–Albus–Ito theory gradually (8–10). Furthermore, researchers have examined the learning theory using realistic spiking network models of the cerebellum (11–17). Especially, Hausknecht et al. (13) examined the learning capability of the cerebellum through a cerebellar spiking network modeled in an SL context. The network successfully performed SL and control tasks but failed to solve RL and complex recognition tasks. Geminiani et al. (17) implemented a spiking network-based cerebellar model featuring bidirectional plasticity at PF–PC and PF–molecular layer interneurons (MLIs) synapses across multiple microzones, with several microzones more likely depression and others more likely potentiation during motor control. Their results highlight the cerebellum’s capacity for error correction and adaptive learning, particularly within the context of associative learning and motor control. Both computational studies modeled the cerebellum as a conventional associative learning machine and highlighted the cerebellar learning capability, especially in the SL context.

One of the other research directions is to adapt reinforcement learning (RL) theory (18) as an alternative to conventional SL. In an RL context, there is an “agent” and an “environment.” The agent takes an action affecting the state of the environment. In response to the action, the environment sends a “reward” to the agent which represents how good/bad the action was. The agent learns appropriate actions that maximize an expected future reward. One of the most notable differences between SL and RL is feedback information from the environment. An SL machine receives errors or teacher signals representing optimal actions, while an RL machine receives only reward signals.

Swain et al. (19) reported that the cerebellum fulfills three key requirements to establish reinforcement learning: it receives sensory information about the external environment and the internal environment, selects a behavior to execute, and processes evaluative feedback on the success of that behavior. Building on this idea, Yamazaki and Lennon (20) proposed the concept that the cerebellum might perform RL. Specifically, CFs deliver reward information, PCs select actions, and MLIs evaluate the current state. Synaptic weights at PF–MLI as well as PF–PC synapses are modulated by the CF signals. The researchers interpreted this structure as an RL machine, specifically as an actor-critic model (18). However, there has been no detailed spike-based implementation for the cerebellar RL concept.

In this research, based on a continuous time actor-critic framework with spiking neurons (21), we implemented a cerebellar spiking network as an actor-critic model based on the known anatomical properties of the cerebellum. In particular, we modified a weight-updating rule of the framework for the cerebellum. We evaluated the model using a simple machine learning benchmark task, known as the mountain car task (22). We also examined the behavior of the model using delay eyeblink conditioning (23), which is a standard cerebellum-dependent motor learning task. To our knowledge, the model presented here is the first cerebellar spiking network model that can act as an RL machine.

## Results

### Spike-based implementation of cerebellum-style RL

**Fig. 1.**
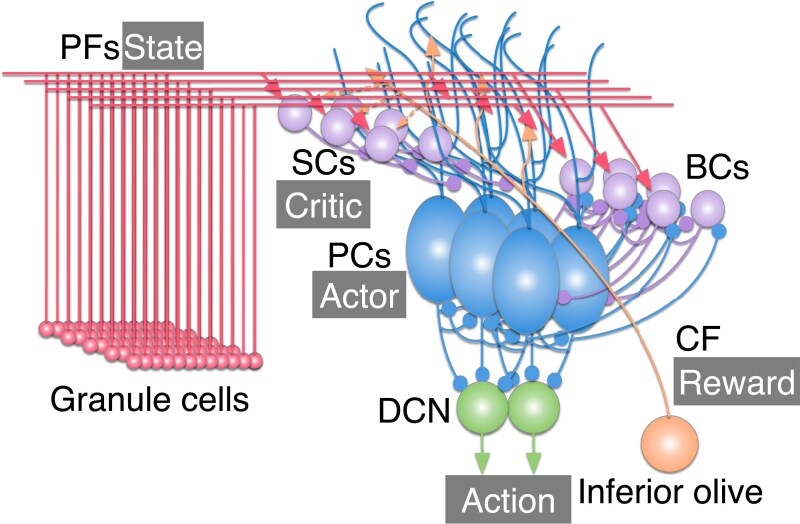
Structure of the present cerebellar network implemented as an actor-critic model. The network consists of granule cells, molecular layer interneurons (SCs and BCs), PCs, DCN, and inferior olive. PFs deliver state information, CF delivers reward information, PCs serve as the actor, and SCs serve as the critic. Triangle-headed arrows and circle-headed arrows represent excitatory synapses and inhibitory synapses, respectively. Dashed lines represent excitatory signals from CF via glutamate spillover (63).

In this research, we implemented a cerebellar spiking network model as an RL machine (Fig. 1) while referring to the cerebellar RL concept (20) and the spiking actor-critic framework (21). Parallel fibers (PFs) excite stellate cells (SCs), basket cells (BCs), and Purkinje cells (PCs). SCs and BCs inhibit PCs. BCs and PCs inhibit each other, and also both have a self-inhibitory connection. More specifically, BCs and PCs are divided into multiple groups, which correspond to action choices as described in Actor and action selection section. BCs inhibit PCs in the same group and BCs in other groups, whereas PCs inhibit BCs in the same group and PCs in other groups. PCs also inhibit neurons in the deep cerebellar nuclei (DCN), which are also divided into multiple groups. CFs provide excitatory inputs to SCs and PCs. We used CF signals to trigger synaptic plasticity. Therefore, we did not implement actual CF–SC and CF–PC connections.

In the cerebellar spiking network model, we implemented an RL algorithm known as the actor-critic model (18) based on the previous spiking actor-critic framework (21). In RL, an agent in state s(t) at time *t* takes action a(t) following a policy *π*. In response to action a(t), the environment changes its state and returns a reward r(t) to the agent. The agent learns to find the optimal policy that maximizes the expected future reward. Specifically, the agent of the actor-critic model consists of two modules: an actor and a critic. The actor calculates preferences of actions $h_{\alpha }(t)$ and decides on action a(t), while the critic computes the state value V(t) which refers to the expected cumulative future reward. The temporal difference error (TD error) is calculated from the returned reward and the state value for updating the inner parameters of both the critic and the actor.

In our implementation, s(t) was transmitted through PFs to PCs, BCs, and SCs. The actor and the critic were PCs and SCs, respectively. Practically, the activity of PCs represented the avoidance of actions $-h_{\alpha }(t)$, while the activity of SCs represented the state value with the inverted sign −V(t) rather than TD error which was represented by MLIs in a previous theoretical model (20). BCs contributed to action selection by modulating the activity of PCs (24, 25). Then, the most activated DCN group decided on an actual action a(t). In response to the action, reward information was transmitted through CFs to PCs and SCs.

#### Representation of states and rewards

States were represented by PFs following the formulation in the classical Marr–Albus–Ito model (1–3). For simplicity, we arranged the PFs on a 2D grid plane composed of a number of small tiles (26). We named this plane the PF plane. Each tile had an individual receptive field that determined the PF’s firing rate based on the observed state, as described in Implementations section.

In the present study, rewards r(t) were actually considered as punishments so that r(t)≤0. To represent this negative reward signal in the spike-based framework, CFs were modeled as a spike train scaled by a task-specific constant as follows:


(1) $$\text{CF}(t)=-\bar{r}S_{\mathrm{CF}}(t),$$

where $\bar{r}$ is a task-specific negative constant and $S_{\mathrm{CF}}(t)$ represents the spike train of the CF. In response to the action, the environment modulates the firing rates of CFs.

#### Critic

We designed the temporal activity of SCs to approximate the state value. Strictly speaking, the state value with the inverted sign was calculated by SCs as follows:


(2) $$-V(t)=\nu \text{SC}(t)-V_{0},$$

where *ν*, SC(t), and $V_{0}$ represent a scaling factor, the temporal activity of SCs, and the baseline of the state value, respectively. The baseline $V_{0}$ was set at *ν* times the average value of SC(t), which was calculated during the latter half of an episode without learning. SC(t) was calculated as follows:


(3) $$\text{SC}(t)=\frac{1}{N_{\mathrm{SC}}}\sum_{i}{\text{SC}}_{i}(t),$$

(4) $${\text{SC}}_{i}(t)=\frac{{\text{SC}}_{i,\text{decay}}(t)-{\text{SC}}_{i,\text{rise}}(t)}{\tau_{\mathrm{SC},\text{decay}}-\tau_{\mathrm{SC},\text{rise}}},$$

(5) $$\frac{d{\text{SC}}_{i,l}(t)}{dt}\;=-\frac{{\text{SC}}_{i,l}(t)}{\tau_{\mathrm{SC},l}}+S_{i}(t),$$

where $N_{\mathrm{SC}}$ and ${\text{SC}}_{i}(t)$ represent the number of SCs and the temporal activity of *i*th SC, respectively. *l* is a label in {rise,decay}. The temporal activity of *i*th SC rises with a time constant $\tau_{\mathrm{SC},\text{rise}}$ and decays to 0 with a time constant $\tau_{\mathrm{SC},\text{decay}}$. Spike train of the *i*th neuron is defined as $S_{i}(t)=\sum_{f}\delta_{D}(t-t_{i}^{\left(f\right)})$ with the Dirac delta function $\delta_{D}(t)$ and the *f*th spike of the *i*th neuron.

#### Actor and action selection

PCs performed the actor and determined the action taken (27). We interpreted the temporal activity of PCs as expressing avoidance of certain actions. Specifically, the PCs in our model compute the avoidance of an action $h_{\bar{\alpha }}(t)$ instead of the preference for an action $h_{\alpha }(t)$. Therefore, the taken action a(t) was determined by the least-avoided action in action space A at time *t*.


(6) $$a(t)=\underset{\alpha \in A}{\text{argmin}}\;h_{\bar{\alpha }}(t).$$

The avoidance of action α∈A was represented by the activity of PCs in $\bar{\alpha }$ group as follows:


(7) $$h_{\bar{\alpha }}(t)=\frac{1}{N_{{\mathrm{PC}}_{\bar{\alpha }}}}\sum_{i\in {\text{PC}}_{\bar{\alpha }}}{\text{PC}}_{i}(t),$$

where $N_{{\mathrm{PC}}_{\bar{\alpha }}}$ is the number of neurons in the group ${\text{PC}}_{\bar{\alpha }}$. ${\text{PC}}_{i}(t)$ was the temporal activity of *i*th PC defined as follows:


(8) $${\text{PC}}_{i}(t)\;=\frac{{\text{PC}}_{i,\text{decay}}(t)-{\text{PC}}_{i,\text{rise}}(t)}{\tau_{\mathrm{PC},\text{decay}}-\tau_{\mathrm{PC},\text{rise}}},$$

(9) $$\frac{d{\text{PC}}_{i,l}(t)}{dt}\;=-\frac{{\text{PC}}_{i,l}(t)}{\tau_{\mathrm{PC},l}}+S_{i}(t),$$

where *l*, $\tau_{\mathrm{PC},l}$, and $S_{i}(t)$ represent the label l∈{decay,rise}, the time constant for *l*, and the spike train of the *i*th PC, respectively. Practically, the actual action was generated at the downstream DCN (see Implementations section).

Those spike activities of PCs were used for updating synaptic weights to realize which action was selected. It was vital that one PC group pauses while the other PC groups activate. We referred to this pausing behavior as a “dent,” while activation of neurons in a specific area was referred to as a “bump” by Frémaux et al. (21). The inhibitory loop between BCs and PCs helped to pause the single group solely to make a dent. Although the original spiking actor-critic framework supports continuous action space, the present model only supports a discrete action space with two actions to demonstrate that the present model can solve tasks in the simplest settings.

#### Weight-update rules

Our weight-update rule was based on TD long-term potentiation (LTP) which had been proposed in a previous spiking actor-critic model (21). However, unlike the previous model, which calculated the time derivative of the state value directly to compute the TD error, our model did not calculate it, as there was no component in the molecular layer of our model that explicitly handled the TD error.

To address this problem, we conducted an eligibility trace and transformed the weight-update rule. As a result, our weight-update rule for the state value was as follows:


(10) $$\frac{d\Delta w_{i,j}^{n}}{dt}\;=\alpha \left(R(t)z_{i,j}(t)+\frac{\tau_{r}-\tau_{z}}{\tau_{r}\tau_{z}}V(t)z_{i,j}(t)-V(t)f_{i,j}(t)\right),$$

(11) $$\frac{dz_{i,j}(t)}{dt}\;=-\frac{z_{i,j}(t)}{\tau_{z}}+f_{i,j}(t),$$

(12) $$f_{i,j}(t)\;=S_{i}(t)\kappa_{i,j}(t),$$

(13) $$\frac{d\kappa_{i,j}(t)}{dt}\;=-\frac{\kappa_{i,j}(t)}{\tau_{\kappa }}+S_{j}(t),$$

where $\Delta w_{i,j}^{n}$, *α*, R(t), $z_{i,j}(t)$, $\tau_{r}$, $\tau_{z}$, $f_{i,j}(t)$, $S_{i}(t)$, $S_{j}(t)$, $\kappa_{i,j}(t)$, and $\tau_{\kappa }$ are the amount of synaptic change in *n*th episode, the learning rate, the negative reward, the eligibility trace for $w_{i,j}$, the reward discount time constant, the decay time constant of eligibility trace, the forcing term of eligibility trace, the postsynaptic spike train, the presynaptic spike train, the window function for spike timing dependent plasticity, and the decay time constant of the window function. According to the previous model (21), the eligibility term memorizes the history of inputs before the time of the last postsynaptic neuron spike, $\kappa_{i,j}(t)$ is reset to 0 after the postsynaptic neuron spike. The relationship of our weight-update rule to the original weight-update rule (21, 28) is described in Relationship between our weight-update rule and the previous weight-update rule section of Supplementary information. The synaptic weight $w_{i,j}$ was updated at the end of every episode $T_{\mathrm{end}}$ by $w_{i,j}^{n+1}=w_{i,j}^{n}+\eta \Delta w_{i,j}^{n}(T_{\mathrm{end}})$ with learning rate *η*, and the synaptic weight was bounded between [0,1]. Since SCs and CFs represented the state value with the inverted sign and the negative rewards, respectively, the equation for the PF–SC synapses was expressed as follows:


(14) $$\frac{d\Delta w_{i,j}^{n}}{dt}\;=\text{CF}(t)z_{i,j}(t)-\frac{\tau_{r}-\tau_{z}}{\tau_{r}\tau_{z}}V(t)z_{i,j}(t)+V(t)f_{i,j}(t).$$

On the other hand, the weight-update rule for PF–PC synapses was expressed as follows:


(15) $$\frac{d\Delta w_{i,j}^{n}}{dt}\;=-\text{CF}(t)z_{i,j}(t)+\frac{\tau_{r}-\tau_{z}}{\tau_{r}\tau_{z}}V(t)z_{i,j}(t)-V(t)f_{i,j}(t).$$

The specific learning rates for PF–SC synapses and PF–PC synapses are denoted as $\eta_{\mathrm{SC}}$ and $\eta_{\mathrm{PC}}$, respectively.

Specifically, given that the conjunctive activation of PFs and CFs induces LTP at PF–SC synapses (29–32), our SCs learned the approximate the state value with the inverted sign. LTD at PF–SC synapses was due to PF firing and was modulated by postsynaptic activation results in bidirectional synaptic plasticity (29–32). At PF–PC synapses, the conjunctive activation of PFs and CFs induces LTD (7), while PF activation induces LTP. These synaptic plasticities are also modulated by inhibitions of SCs (33), and we assumed that PC activity contributes to eligibility trace and bidirectional synaptic plasticity (34, 35).

Additionally, we confirmed that our critic is capable of learning state values through a Linear track task simulation (see Simulation of linear track task section of Supplementary information), which is a simplified version of the water-maze task used to isolate learning by the critic from that by the actor (21).

### Simulation of mountain car task

**Fig. 2.**
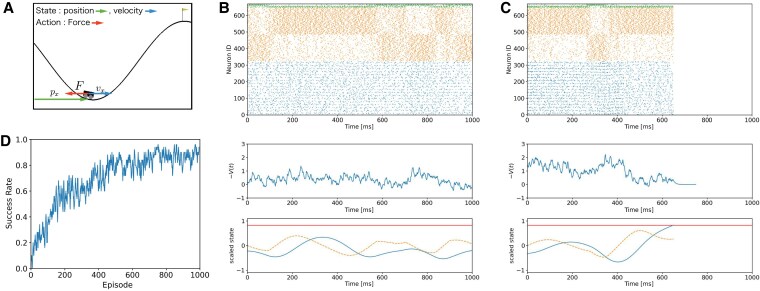
Simulation of the mountain car task. A) Schematic of the mountain car task. $p_{x}$ is the horizontal position of the cart, $v_{x}$ is the horizontal velocity, and *F* is the external force applied to the cart by the agent. The flag marks the goal position. For clarity in the image, we adjusted the *x*-axis to start at 0 on the left edge. B, C) Comparison of behaviors in the mountain car task between (B) an early failure episode and (C) a later successful episode. Top panels show raster plots of SCs (0–323), BCs (324–647) and PCs (648–667). The number of these neurons are 324, 324, and 20, respectively. Middle panels show a trajectory of −V(t) computed from the activity of SCs. Bottom panels show trajectory of scaled states. Blue solid line and orange dashed line represent the normalized value of a position and that of a velocity. Horizontal red solid line represents the goal position. When the position reaches the goal, an episode ends while a simulation lasts 100 ms (see Pre- and postepisode processes section). D) Change of the success rate.

In order to evaluate the learning capability of our model as an RL machine, we applied our model to the mountain car task (36), a classic RL challenge, and evaluated its performance over independent 10 runs, each learning for 1,000 episodes. In this task, an agent controls a car situated between two hills (Fig. 2A). The goal is to have the car climb up the mountain to reach a flag positioned at the peak of the right hill. Due to the car’s limited engine power and the hill’s steepness, direct ascent is not possible. An agent observes a position $p_{x}(t)$ and a horizontal element of the car velocity $v_{x}(t)$, and decides the direction (either left or right) to push the car with a constant force $F(t)=\bar{F}a(t),a(t)\in \{-1,1\}$. In early episodes, the car was confined to moving at the bottom of the valley, and failed to reach the goal (Fig. 2B). However, after hundreds of episodes, our model learned to utilize the slope for acceleration, and eventually reached the goal (Fig. 2C). Specifically, we found that the trajectory involved the car ascending the right hill, then the left, and finally the right hill again. The state value with the inverted sign −V(t), which was approximated by SC activity, was noisy but mostly stayed around 0 at every state before learning (Fig. 2B). In a successful episode after learning (Fig. 2C), the value was high at the start and decreased over time as the car approached the goal. The moving average of success rate over 10 runs progressively increased and stabilized at ∼80% after 600 episodes (Fig. 2D).

These results indicate that our model solved the mountain car task, and suggest that our model successfully acted as an RL machine.

### Simulation of delay eyeblink conditioning task

**Fig. 3.**
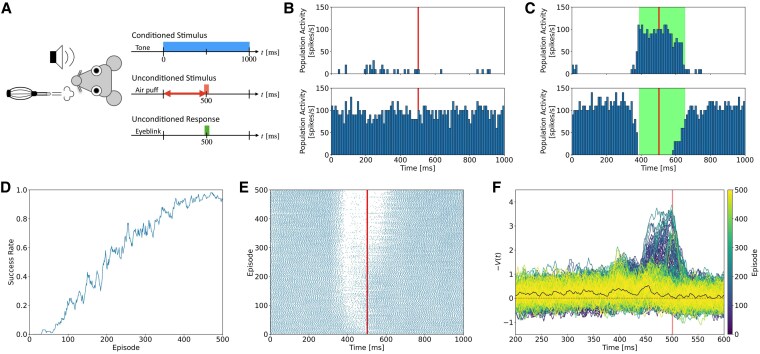
Simulation of the delay eyeblink conditioning task. A) Schematic of the task. A neutral stimulus (CS) is presented, followed by an aversive stimulus (US) after a fixed interval (t=500ms). After repeated pairings, the agent closes its eyes (CR) in response to the CS alone. B, C) Population activity of PCs during (B) a failure episode and (C) a success episode. Top and bottom panels show the population activity of antiopen and anticlose PC populations, respectively. Bin width is 10 ms. US onset is indicated by a vertical red line. The period during the agent selects the eye-closing action is shown by green shaded rectangle. D) Change of the success rate. E) The activity changes of a single PC in the anticlose group, with dots representing spikes and a red line indicating US onset. F) Change of the value of −V(t) computed from the activity of SCs across episodes. The color of each trace indicates the episode number, ranging from 0 to 500, as shown by the color bar. The black bold line represents the average over the last 10 episodes, the brown dashed line shows −V(t)=0, and the vertical red line indicates US onset.

To examine whether our cerebellar model shows internal dynamics consistent with the biological cerebellum, we conducted simulation of a standard cerebellum-dependent motor learning task known as the delay eyeblink conditioning task (23) over 10 runs, each learning for 500 episodes. In this task (Fig. 3A), an animal is presented with two types of stimuli: a conditioned stimulus (CS) and an unconditioned stimulus (US). The CS, a neutral stimulus such as a tone, initially does not trigger any response. On the other hand, the US, such as an air puff, naturally induces an unconditioned blink reflex in the animal. However, when the CS is repeatedly paired with the US, the animal begins to anticipate the air puff whenever the CS is presented. This anticipation leads the animal to blink in response to the CS alone, a learned behavior known as the conditioned response (CR). We modified the task to have 2D state space: time *t* and eyelid position $p_{e}(t)$, along with two actions: opening and closing the eyelid. The time axis ranges from 0 ms to 1,000 ms, corresponding to the duration of stimulus presentation, while the eyelid position is measured on a scale from fully closed (0) to fully open (1). The CS was presented from 0 ms to 1,000 ms as a time signal, and the US was introduced at 500 ms as a punishment if the eyelid was open, i.e. if $p_{e}(500)$ was greater than 0.1.

In early episodes (Fig. 3B), the agent preferred to open the eyelid, so the agent received the US as a negative reward. As learning progressed (Fig. 3C), the antiopen PC group became active, whereas the anticlose PC group paused as if PCs expected the US onset. Thus, the eyelid started to close before the US onset. For every 10 runs, the agent successfully acquired the CR to the CS (Fig. 3D): closing its eyelid to avoid a punishment before the arrival of the US. Note that we defined a successful episode as a sufficiently closed eyelid to avoid the punishment at the US onset. When focusing on the activity changes of a single PC in the anticlose group (Fig. 3E), the neuron acquired a behavior to pause before US onset. These behaviors were consistent with behavioral experiments of the delayed eyeblink conditioning task (23).

We also analyzed the activity of SCs during the task (Fig. 3F). In early episodes, since the agent received the punishment as the US, the SC activity as the state value with the inverted sign −V(t) ramped up toward the arrival of the US. These results are not inconsistent with biological experiments (37, 38), which show increases in MLIs’ firing frequency from the time of CS onset to the time of US onset. In later episodes when the agent successfully closed the eyelid more than 80% of the time, the SC activity returned to almost baseline. This returning behavior resulted from the disappearance of CF signals due to the successful avoidance of punishments.

**Fig. 4.**
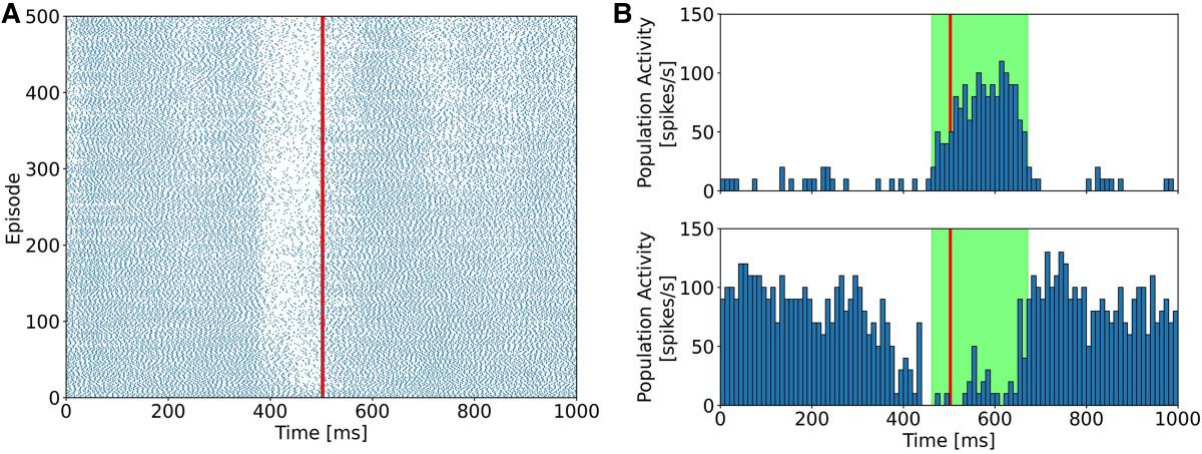
Results of the delay eyeblink conditioning task without PF–SC synaptic plasticity. A) The activity changes of a single PC in the anticlose group. Dots represent spikes of the PC, and vertical red line represents the time of US onset. B) Example PC population activity of (top) antiopen PC population and (bottom) those of anticlose PC population in a later episode. Bin width is 10 ms. Vertical red lines represent the time of US onset, while the green shaded rectangles show when the agent closes its eyelid around the US onset.

We also conducted a simulation of the same task without PF–MLI synaptic plasticity. The activity of the anticlose PC group decreased as it approached the US onset (Fig. 4A). However, the agent was not able to elicit a stable closing response in our settings. In a later episode, the time to close the eye was delayed (Fig. 4B) compared to that in the intact simulation (Fig. 3C), so the agent could not close the eye sufficiently.

These results are also consistent with experimental results where MLI–PC inhibitions were impaired (39). Furthermore, these results suggested that our model successfully solved the eyeblink task with biologically plausible internal dynamics.

## Discussion

In this study, we implemented a cerebellar spiking network model performing RL in an actor-critic manner. Specifically, we interpreted that PFs and CF represent states and negative reward signals, respectively. SCs act as a critic and represent the state value with the inverted sign, while PCs act as an actor and represent avoidance of certain actions. Our simulations confirmed the model’s ability to solve a simple RL task and demonstrated a cerebellum-dependent motor leaning task with biologically plausible internal dynamics.

In a standard RL paradigm, a value called a TD error or a reward prediction error (RPE), which represents the difference between an actual reward and a predicted reward, plays an essential role. In the cerebellum, Ohmae and Medina (40) first reported RPE-like complex spike activities in PCs during eyeblink conditioning in mice. After the pioneering work, experimental studies in mice demonstrated that CFs carry multiplex information including not just motor-related information but also reward-related information such as reward delivery, prediction, and omission (41–45). Hoang et al. (46) recapitulated the idea of RPE conveyed by CFs to Q-learning. While these studies interpreted that RPEs are provided by CFs, another hypothesis is that CFs convey negative reward information, and MLIs compute RPE. This hypothesis is more closely related to conventional motor adaptation studies of the cerebellum, because negative reward information could be regarded as a form of teaching signals. Experimental findings about the functional roles of MLIs beyond MLI–PC inhibition (24, 25, 32, 47–50), assistive roles of MLIs in cerebellar learning (37–39, 51), plasticity at PF–MLI synapses (29–31), and the modulatory role in PF–PC synaptic plasticity (33) supports the hypothesis. According to these studies, Yamazaki and Lennon (20) interpreted that MLIs provide RPE and proposed a conceptual cerebellar RL model as an actor-critic model.

The present study follows the latter hypothesis, because our model interpreted the SC population as a critic. On the other hand, by introducing our learning rule as in (14) and (15), our model does not need to calculate TD errors explicitly. Even without the explicit calculation, our model succeeded in solving the simple RL task and also suggested that impairments in PF–SC synaptic plasticity may affect the stability of movement acquired through an association task. With the new learning rule, first, SC population activity gradually increased toward the onset of the CF signal (Figs. 3F and S1B), and then the SC activity returned to baseline once the optimal policy was acquired and no further punishment was expected (Fig. 3F). These observations imply that there is no way to distinguish the activities of sufficiently trained SCs with those of untrained SCs. To observe a learning-dependent transient increase/decrease of SC activities, one needs to track the activities of SCs persistently during the whole period of a behavioral training. It is possible to track the same granule cells over several days of learning (52). Similar attempts could be made to SCs.

Although the present study proposes that the cerebellum can act as an RL machine, this proposal does not necessarily deny the classical and standard view of the cerebellum as an SL machine. Rather, we consider that both SL and RL co-exist within the cerebellum. There are two possibilities for the co-existence. First, a single CF could deliver both evaluative and teaching signals for RL and SL, respectively, thereby a single microzone can perform both RL and SL simultaneously. In this scenario, teaching signals mainly drive learning, while evaluative signals may play an assistive role for the learning. By combining teaching signals with evaluative signals, the cerebellum may learn appropriate actions more quickly than when using evaluative signals alone. Second, a single CF innervating a microzone delivers either evaluative or teaching signals, thereby a single microzone can perform either RL or SL, and multiple microzones participate to perform both RL and SL simultaneously. In this scenario, distinct microzones may specialize in different learning strategies, some implementing RL and others SL, depending on their functional roles. In both scenarios, by combining SL and RL, the learning capability of the cerebellum can be enhanced for faster, smoother, and more complex motor and cognitive tasks such as realtime motor control and online learning (14, 53–55).

Our model still has several limitations. First, we limited actions to two discrete ones. In our model, PCs directly inhibited but indirectly disinhibited each other via BCs to form a single dent. On the other hand, in the previous spiking actor-critic framework (21), actor neurons that directly inhibit and excite each other to form a single bump and employ population vector coding. Therefore, for a broader action space, more precise parameter adjustment of the intra-layer synaptic connections of the actor in our model would be necessary to implement population vector coding in PCs. Another limitation of our model is that the CF activity was modeled as a spike train. To represent negative rewards, only the firing rate of CF was controlled in each task within the biologically plausible firing rate. This limitation may spoil the learning performance of our model in more complex RL tasks. However, recent studies (56–58) have showed that CF signals can carry graded information. Adopting the mechanisms might enhance the learning performance of our model in the tasks.

If both the cerebellum and the basal ganglia perform RL, how do these regions cooperate for learning? One possible interpretation is hierarchical RL (HRL) (18) as discussed in our previous research (10). HRL considers two RL machines organized hierarchically. The higher RL machine breaks down a complex task into simpler, smaller subtasks. The lower RL machine tries to solve these subtasks one by one. Eventually, HRL involves creating a hierarchy of policies that makes the learning process more efficient and scalable (18, 59, 60). If the basal ganglia and the cerebellum cooperate to perform HRL, the resulting network would demonstrate powerful learning capabilities.

To our knowledge, our model is the first to implement general cerebellar RL with spiking neurons. Our research provides an implementation that supports cerebellar RL theory to challenge the traditional view of the cerebellum as primarily a SL machine. Furthermore, our interpretation of a potential cooperative role of the basal ganglia and the cerebellum as HRL sheds light on how the multiple brain regions act together synergistically as a whole.

## Materials and methods

### Implementations

First, we discretized a state space and mapped it on a PF plane with grid size $N_{x}\times N_{y}$. A tile positioned at (i,j) on the PF plane had its own responsible state $o_{i,j}$ as follows:


(16) $$o_{i,j}=[i\Delta s_{x},j\Delta s_{y}{]}^{T},$$

(17) $$\Delta s_{x}=\frac{s_{x,\max}-s_{y,\min}}{N_{x}},\;\Delta s_{y}=\frac{s_{y,\max}-s_{y,\min}}{N_{y}},$$

where *x* and *y* are the axes of the PF plane, and $\left(s_{x,\min},s_{x,\max}\right)$ and $\left(s_{y,\min},s_{y,\max}\right)$ represent the ranges of state *x* and *y*, respectively. When the agent observed state $o(t)=[x(t),y(t){]}^{T}$, PFs on a tile at (i,j) fired as an inhomogeneous Poisson spike generator with a firing rate $\rho_{i,j}(o(t))$ defined as follows:


(18) $$\rho_{i,j}(o(t))=\;\frac{\rho_{\mathrm{PF}}}{\sqrt{2\pi \sigma^{2}}}\exp\;\left(-\frac{\|o(t)-o_{i,j}{\|}^{2}}{2\sigma^{2}}\right),$$

where $\rho_{\mathrm{PF}}=50\;\text{Hz}$ is a scaling factor of firing rate, and σ=0.5 is a standard deviation. To maintain the total activity of all PFs when the agent is at the edge of a state space, we added “margin” tiles as an outer frame. Although we modeled PFs as Poisson spike generators for simplicity, more realistic implementations are reported (61).

We utilized a leaky integrate-and-fire model (LIF) to SCs, BCs, and PCs. A LIF model was implemented as follows.


(19) $$C_{m}\frac{du_{i}}{dt}\;=-g_{L}(u_{i}-E_{L})+I_{i,\text{syn}}(t)+I_{\mathrm{ext}}+I_{i,\text{noise}}(t),$$

(20) $$t_{i}^{\left(f\right)}\;:\;u_{i}\left(t_{i}^{\left(f\right)}\right)=U_{\mathrm{th}},$$

(21) $$\lim_{t_{i}^{\left(f\right)}\to t,\;t>t_{i}^{\left(f\right)}}u_{i}(t)=U_{\mathrm{reset}},$$

where $C_{m}$, $u_{i}$, *t*, $g_{L}$, $E_{L}$, $I_{i,\text{syn}}$, $I_{\mathrm{ext}}$, and $I_{i,\text{noise}}$ represent the membrane capacitance, the membrane potential of the *i*th neuron, time, the leak conductance, the resting potential, the synaptic current to the *i*th neuron, the external input current, and the noise current to the *i*th neuron, respectively. When the membrane potential of the *i*th neuron reaches the threshold potential $U_{\mathrm{th}}$, that time is denoted as $t_{i}^{\left(f\right)}$, and the neuron fires the *f*th spike and resets its membrane potential to the reset potential $U_{\mathrm{reset}}$. A synaptic current was defined as follows:


(22) $$I_{i,\mathrm{syn}}(t)=\sum_{c}s_{c}w_{i,j}I_{i,j}(t),$$

(23) $$\frac{dI_{i,j}(t)}{dt}\;=-\frac{I_{i,j}(t)}{\tau_{c}}+S_{j}(t),$$

where *c* is a synapse label, $s_{c}$, $w_{i,j}$, $\tau_{c}$, and $S_{j}(t)$ are the scaling factor, synaptic weight between the *j*th presynaptic neuron and the *i*th postsynaptic neuron, decay time constant, and spike train of the *j*th presynaptic neuron, respectively. Whether it is an inhibitory or excitatory current is determined by the sign of the scaling factor. Noise current $I_{i,\text{noise}}(t)$ was modeled as follows:


(24) $$\frac{dI_{i,\text{noise}}}{dt}\;=-\frac{I_{i,\text{noise}}}{\tau_{\mathrm{noise}}}+\zeta n(t),$$

where $\tau_{\mathrm{noise}}$ is a time constant, and *ζ* is an amplitude factor. n(t) is a uniform random variable in the range [−1,1]. Parameters of all neurons were set as listed in Table 1, and parameters of all synapses were set as listed in Table 2.

**Table 1.** Neuron-specific parameters.

Type	$C_{m}$ [pF]	$g_{L}$ [mS]	$E_{L}$ [mV]	$U_{th}$ [mV]	$U_{r}$ [mV]	$I_{ext}$ [pA]	*ζ* [pA]
SC	107	2.32	−68.0	−55.0	−70.0	30.0	5.0
BC	107	2.32	−68.0	−55.0	−70.0	30.0	1.0
PC	107	2.32	−65.0	−55.0	−66.0	30.0	5.0

These parameters were adjusted from those used in our previous model (15).

**Table 2.** Synaptic parameters for each connection type.

Connection type	Scaling factor	Weight	$\tau_{c}$ (ms)	Probability
PF–SC	200.0	–	8.3	0.1
PF–BC	10.0	1.0	8.3	0.5
PF–PC	5.0	–	8.3	0.5
SC–PC	−0.05	0.03	10.0	0.5
BC–BC	−2.0	1.0	10.0	0.3
BC–PC	−1.0	1.0	10.0	0.3
PC–BC	−2.0	1.0	10.0	0.8
PC–PC	−19.8	1.0	5.0	1.0

A hyphen in a weight cell means it will be modified because of synaptic plasticity.

As mentioned above, PFs were distributed on the 2D grid plane, and the grid size was determined by each task. Each tile contained 100 PFs. SCs were prepared 1 per tile. Each SC positioned at $\left(i\Delta s_{x},j\Delta s_{y}\right)$ could receive excitatory inputs from PFs located in $s_{x,\min}\leq x\leq s_{x,\max}$ and $(j-1)\Delta s_{y}\leq y\leq (j+1)\Delta s_{y}$. The number of BCs was the same as that of SCs, and the number of PCs was 20. Both BCs and PCs could receive excitatory PF inputs from all tiles. The BC–BC, BC–PC, PC–BC, and PC–PC connections were constructed based on the grouping manner mentioned in Spike-based implementation of cerebellum-style RL section. The actual synapses were formed stochastically (Table 2). In this study, we divided BCs and PCs into two groups for all tasks. Additionally, for the sake of simplicity, we assumed that all CFs synchronized their activity so that our model had a single CF.

Synaptic plasticity was applied to PF–SC and PF–PC synapses. The details of the weight-update rules are described in (14) and (15).

Finally, the outcome of actions were generated by DCN cells prepared in an equal number of PCs. Each DCN cell was inhibited by its counterpart PC. This configuration means that the activity of each DCN cell, denoted as ${\mathrm{DCN}}_{i}$, was modulated by the corresponding PC activity ${\mathrm{PC}}_{i}(t)$ defined by Equation 8, as follows:


(25) $$\frac{d{\mathrm{DCN}}_{i}(t)}{dt}\;=-\frac{{\mathrm{DCN}}_{i}(t)-{\mathrm{DCN}}_{0}}{\tau_{\mathrm{DCN}}}-\nu {\mathrm{PC}}_{i}(t),$$

where $\tau_{\mathrm{DCN}}$, ${\mathrm{DCN}}_{0}$, and *ν* represent the decay time constant, baseline activity of DCN, and a scaling factor, respectively.

Since PCs in the group ${\mathrm{PC}}_{\bar{\alpha }}$ represented the avoidance of action *α* and inhibited DCN cells, the activity of DCN group ${\mathrm{DCN}}_{\alpha }$ represented the preference of actions $h_{\alpha }(t)$ as follows:


(26) $$h_{\alpha }(t)=\;\frac{1}{N_{{\mathrm{DCN}}_{\alpha }}}\sum_{i\in {\mathrm{DCN}}_{\alpha }}{\mathrm{DCN}}_{i}(t),$$

where $N_{{\mathrm{DCN}}_{\alpha }}$ is the number of neurons in the group ${\mathrm{DCN}}_{\alpha }$, and ${\mathrm{DCN}}_{i}(t)$ is the activity of *i*th DCN in the group ${\mathrm{DCN}}_{\alpha }$ corresponding to action *α*. Therefore, the most activated DCN group represented the most preferred action so that the final outcome of actions was chosen as follows:


(27) $$a(t)=\underset{\alpha \in A}{\text{argmax}}\;h_{\alpha }(t).$$

### Task definitions

#### Pre- and postepisode processes

As a pre-episode process, at the start of each episode, a free run for 200 ms was carried out to discard initial transient activity that could affect learning. Then, at the end of each episode, a simulation continued for 100 ms while stopping the firing of PFs to update synaptic weights. These pre- and postepisode processes were performed in all tasks.

#### Mountain car task

The dynamics of the car were defined by the following differential equations:


(28) $$\frac{dv_{x}(t)}{dt}\;=v_{x}(t)+\bar{F}a(t)-g\cos\;\left(3p_{x}(t)\right)$$

(29) $$\frac{dp_{x}(t)}{dt}\;=v_{x}(t),$$

where $\bar{F}=0.001$ is a force amplitude, a(t)∈{−1,1} represents an action as the force direction, g=0.0025 represents the gravity. Observation spaces of the velocity and position are bounded by [−1.2,0.6] and [−0.07,0.07], respectively. When the car collides with the wall, its velocity was immediately reset to 0 without any negative reward associated with the collision. In the original settings, an agent receives constant negative reward at every step. However, the maximum firing frequency of CF, which is approximately 10 Hz (62), is not enough to represent the constant negative reward. Therefore, in this study, we modeled a new negative reward function for CF. CF fired when the car was far from the goal and the speed was slow. Thus, the firing rate of CF ν(t) was defined as follows:


(30) $$\nu (t)=\bar{\nu }\left(1-\frac{\;p_{\mathrm{goal}}-p_{x}(t)}{p_{\mathrm{goal}}-p_{\mathrm{wall}}}\right)\left(1-\frac{\left|v_{x}(t)\right|}{v_{\max}}\right),$$

where $\bar{\nu }$, $p_{\mathrm{goal}}$, $p_{\mathrm{wall}}$, and $v_{\max}$ are max firing rate of 5 Hz, goal position, left side wall position, and limit of speed respectively. The constant $\bar{r}$ in (1) was set to −5.

The car starts from the bottom of the valley with a velocity of 0 in every episode. The action a(t) represents the direction in which the agent pushes the car. An episode ends when the car reaches the goal or when the elapsed time exceeds 1,000 ms, with no special negative reward given.

The grid size of the PF plane was 16×16. The parameters of the state value function, as defined in (2), were ν=200 and $V_{0}=2.48$. The reward discount time constant $\tau_{r}$, the decay time constant of eligibility trace $\tau_{z}$, and the decay time constant of the window function $\tau_{\kappa }$ were set to 100ms, 20ms, and 20ms, respectively. Initial synaptic weights of PF–SC and PF–PC were 0.05 and 0.8, respectively. Both learning rates for the critic and the agent were 0.4.

We conducted 10 runs of 1,000 episodes each. We calculated the changes in success rate for each run using a moving average of results (success=1, failure=0) with a window size of 10 episodes, and then calculated the average over 10 runs (Fig. 2D).

#### Delay eyeblink conditioning task

The dynamics of the eyelid was modeled as follows:


(31) $$\frac{dp_{e}(t)}{dt}\;=\;0.01a(t),$$

where a(t)∈{−1,1} represents the action the agent takes at time *t*. The CF was assumed to fire once when the air puff was applied to the eye and the eyelid was not closed sufficiently. Thus, the spike train of CF was defined as follows:


(32) $$S_{\mathrm{CF}}(t)=\left\{ \begin{array}{cc} \delta_{D}(t-T_{\mathrm{US}}) & \text{if }p_{e}(t)>0.1,\\ 0 & \text{otherwise}, \end{array}\right.$$

where $T_{\mathrm{US}}=500\;\text{ms}$ represents the US onset. The constant $\bar{r}$ in (1) was set to −5.

The grid size of the PF plane was 16×16. The parameters of the state value function, which defined in (2), were ν=200 and $V_{0}=2.48$. The reward discount time constant $\tau_{r}$, the decay time constant of eligibility trace $\tau_{z}$, and the decay time constant of the window function $\tau_{\kappa }$ were set to 100, 20, and 20ms, respectively. To prioritize the “open” action, we initially set synaptic weights for PF–${\mathrm{PC}}_{\mathrm{anticlose}}$ to 0.8, while PF–${\mathrm{PC}}_{\mathrm{antiopen}}$ was set to 0.7. We conducted 10 runs of the simulation for 500 episodes with learning rates $\eta_{\mathrm{SC}}=0.05$ and $\eta_{\mathrm{PC}}=0.3$. We calculated the changes in success rate for each run using a moving average of results (success=1, failure=0) with a window size of 10 episodes, and then calculated the average over 10 runs (Fig. 3D).

The whole code of our model and all environments were written in C++. All ordinary differential equations were solved numerically with a forward Euler method with a temporal resolution of 1.0 ms.

## Supplementary Material

pgaf302_Supplementary_Data

## Data Availability

The code used in this study, including the implementation of the cerebellar model and the environments, is available at the author’s GitHub repository: https://github.com/Rkuriyama/CeRL.

## Preprints

This manuscript was posted on a preprint: https://doi.org/10.1101/2024.06.23.600300.
