# Supplementary material for: A theory of cerebellar learning as spike-based reinforcement learning in continuous time and space
Source: PNAS Nexus. 2025 Sep 18;4(10):pgaf302. doi: 10.1093/pnasnexus/pgaf302 (PMC12483077; doi:10.1093/pnasnexus/pgaf302)
Supplement: pgaf302_Supplementary_Data [file pgaf302_supplementary_data.pdf]

Supporting Information for

A theory of cerebellar learning as spike-based reinforcement learning in continuous time and space

Rin Kuriyama, Hideyuki Yoshimura, and Tadashi Yamazaki

Tadashi Yamazaki.
E-mail: contact25@numericalbrain.org

This PDF file includes:

Supporting text
Fig. S1
SI References

Supporting Information Text

Relationship between our weight-update rule and the previous weight-update rule

The weight-update rule and eligibility trace in our model are defined as follows:

$$\frac{d\Delta w^n_{i,j}(t)}{dt} = R(t)z_{i,j}(t) + \frac{\tau_r - \tau_z}{\tau_r \tau_z} V(t) z_{i,j}(t) - V(t) f_{i,j}(t), \quad [1]$$

$$\frac{dz_{i,j}(t)}{dt} = -\frac{z_{i,j}(t)}{\tau_z} + f_{i,j}(t). \quad [2]$$

By incorporating the definitions, $\frac{d\Delta w_{i,j}(t)}{dt}$ can be transformed as follows (Equations):

$$\frac{d\Delta w^n_{i,j}(t)}{dt} = R(t)z_{i,j}(t) + \frac{\tau_r - \tau_z}{\tau_r \tau_z} V(t) z_{i,j}(t) - V(t) f_{i,j}(t) \quad [3]$$

$$= R(t)z_{i,j}(t) - \frac{1}{\tau_r} V(t) z_{i,j}(t) + \frac{1}{\tau_z} V(t) z_{i,j}(t) - V(t) f_{i,j}(t) \quad [4]$$

$$= R(t)z_{i,j}(t) - \frac{1}{\tau_r} V(t) z_{i,j}(t) + V(t) \left\{ \frac{1}{\tau_z} z_{i,j}(t) - f_{i,j}(t) \right\} \quad [5]$$

$$= R(t)z_{i,j}(t) - \frac{1}{\tau_r} V(t) z_{i,j}(t) - V(t) \frac{dz_{i,j}(t)}{dt}. \quad [6]$$

The total weight change over an episode $\Delta w_{i,j}(T_{\text{end}})$ is expressed as follows:

$$\Delta w_{i,j}(T_{\text{end}}) = \Delta w_{i,j}(0) + \int_0^{T_{\text{end}}} \frac{d\Delta w^n_{i,j}(t)}{dt} dt \quad [7]$$

$$= \Delta w_{i,j}(0) + \int_0^{T_{\text{end}}} \left\{ R(t)z_{i,j}(t) - \frac{1}{\tau_r} V(t) z_{i,j}(t) - V(t) \frac{dz_{i,j}(t)}{dt} \right\} dt \quad [8]$$

$$= \Delta w_{i,j}(0) - [V(t)z(t)]_0^{T_{\text{end}}} + \int_0^{T_{\text{end}}} \left\{ R(t)z_{i,j}(t) - \frac{1}{\tau_r} V(t) z_{i,j}(t) + \dot{V}(t) z_{i,j}(t) \right\} dt \quad [9]$$

$$= \Delta w_{i,j}(0) - [V(t)z(t)]_0^{T_{\text{end}}} + \int_0^{T_{\text{end}}} \left\{ R(t) - \frac{1}{\tau_r} V(t) + \dot{V}(t) \right\} z_{i,j}(t) dt. \quad [10]$$

Here, by assuming $\Delta w_{i,j}(0) = 0$, $z(0) = 0$, and $V(T_{\text{end}}) = 0$, the following equation is obtained:

$$\Delta w_{i,j}(T_{\text{end}}) = \int_0^{T_{\text{end}}} \left\{ R(t) - \frac{1}{\tau_r} V(t) + \dot{V}(t) \right\} z_{i,j}(t) dt. \quad [11]$$

The expression within the curly brackets is equivalent to the continuous time TD error defined by (1), which was used by the previous spiking actor-critic framework (2).

Simulation of Linear Track Task

In order to evaluate whether our critic could approximate $-V(t)$, we conducted a simulation of the linear track task (2). In this task, the environment is a narrow rectangular plane (Figure S1A), on which the agent consistently moves from the leftmost side (start) to the rightmost side (goal) at a fixed speed. Thus, the agent always reaches the goal at the same time, and receives the same negative reward across all episodes. Once the time constant of the reward discount τ_r is determined, the theoretical value can be solved (Figure S1A).

In this task, the plane size was 40×1, and a fixed starting position was $s = 5$. The agent ran with a fixed speed of 0.05 per ms toward a goal area ($s > 39$). When the agent reached the goal, a negative reward of -5 was given. The parameters of the state value function, which defined in Equation 2, were $\nu = 300$ and $V_0 = 12.4$. The learning rate and the initial weight of PF–SC were 0.04, and 0.05. The reward discount time constant τ_r, the decay time constant of eligibility trace τ_z, and the decay time constant of the window function τ_κ were set to 100 ms, 20 ms, and 20 ms, respectively.

On the first episode, the SC represented a noisy, around zero value function (Figure S1B). Over 100 episodes, the SCs learned the value function progressively. Then the SC activity increased exponentially toward the goal, as if it expected the given negative reward (Figure S1B). The averaged value in 10 late episodes, as plotted with a black line, nicely matches the theoretical value function. These results were consistent with previous research (2), and suggest that our weight-update rules were successful and that the SCs were able to approximate the state value with the inverted sign $-V(t)$.

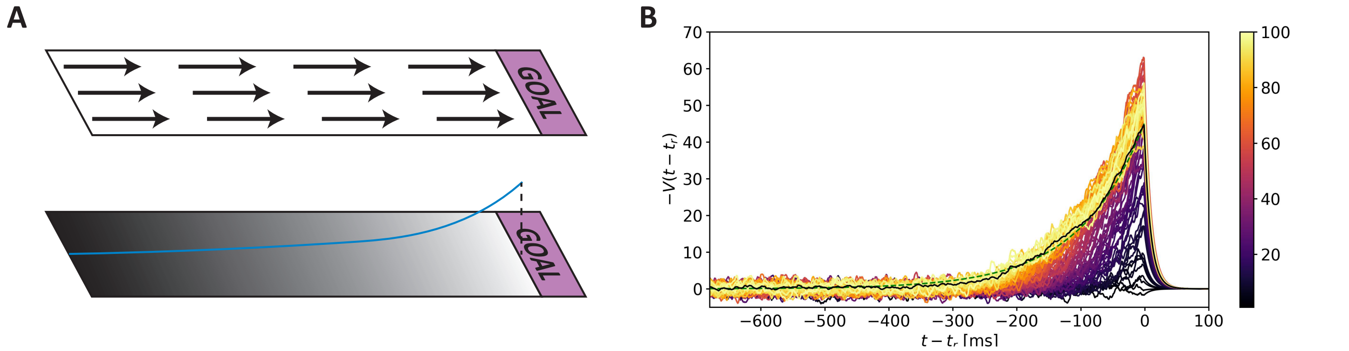

Fig. S1. Simulation of Linear Track Task. (**A**) Environment of the linear track task. Top panel shows the environment and fixed policy. The agent starts at the left edge, and moves straight to the goal shown as a pink area. Bottom panel shows theoretical state value. When the agent follows a fixed policy, the theoretical state value is uniquely determined. (**B**) Change of the value of $-V(t - t_r)$ learned by the critic. Each colored trace shows the value against time in 100 episodes (from dark purple in the 1st episode to light yellow in the 100th episode). The plots are aligned with the timing of the negative reward delivery t_r. The black line shows the average of over last 10 episodes. The green dashed line shows the theoretical value function.

References

1. K Doya, Reinforcement Learning in Continuous Time and Space. *Neural Comput.* **12**, 219–245 (2000).
2. N Frémaux, H Sprekeler, W Gerstner, Reinforcement Learning Using a Continuous Time Actor-Critic Framework with Spiking Neurons. *PLoS Comput. Biol.* **9**, e1003024 (2013).
